# Immunity Enhancement in Immunocompromised Gastrointestinal Cancer Patients with Allogeneic Umbilical Cord Blood Mononuclear Cell Transfusion

**DOI:** 10.1155/2017/5945190

**Published:** 2017-04-26

**Authors:** Ying Qiu, Ruidong Zhao, Mark M. Yun, Xia Han, Feiyu Yun, Bingchun Liu, Erxia Zhou, Xiaohui Ouyang, Sheng Yun

**Affiliations:** ^1^Stem Cell Centre/Department of Oncology, Affiliated Hospital of Inner Mongolia Medical University, Huhhot 010050, China; ^2^Medical School, University College London, Gower Street, London WC1E 6BT, UK

## Abstract

*Objectives*. In order to enhance the immunity of cancer patients to prevent relapse or to prolong survival time, umbilical cord blood mononuclear cells (UCMCs) were transplanted to cancer patients.* Patients and Methods*. UCMCs were transfused to 63 immunocompromised gastrointestinal cancer patients with nonmyeloablative (NMA) conditioning regimen.* Results*. The clinical study showed that the number of both T and B cells increased much more rapidly after transfusion of UCMCs than that of the control group without transplantation (*p* < 0.01). Proinflammation cytokines IFN*γ* and TNF*α* in serum increased to or above the normal range in 80.9% of patients at 12 weeks after UCMC transfusion. However, they recovered to the normal range in 21.7% of patients at the same time point in the control group only. In addition, the clinical investigation also showed that the transfusion of UCMC increased stable disease (SD) and reduced progressive disease (PD) significantly (*p* < 0.01); however, it did not have significant effects on complete response (CR), partial response (PR), or mortality rates compared with the control group (*p* > 0.05).* Conclusions*. UCMCs have powerful repairing effects on damaged cells and tissues and may reconstruct the impaired immunity. Transfusion of UCMCs could reconstruct the immunity of cancer patients with immunosuppression.

## 1. Introduction

Occurrence and progress of cancers are closely related to primary or secondary immunosuppression [1–5]. A variety of chemical and biological factors can cause immune deficiency, such as cytotoxic drugs and viral infections [6, 7]. Malignancies can lead to immune dysfunction or inhibition by direct violation of the immune system. Cancer cells may also suppress host immunity and promote self-progression by releasing immunosuppressive factors or inducing immunosuppressors [8, 9]. Currently, the clinical treatment of gastroenteric malignancies is still dominated by surgical procedures supplemented by chemotherapy and radiotherapy. Although chemotherapy may prolong survival for some cancer patients with disseminated disease, the treatment tends to cause considerable damage to normal cells as well. The most commonly used cytotoxic drugs of chemotherapy in gastroenteric cancers are 5-fluorouracil and platinum, which cause myelosuppression and inhibit lymphocyte proliferation. The damage to immunity may induce cancer cell recurrence and metastasis in cancer patients. Thus, rapidly restoring immunity is one of the key therapeutic strategies to control cancer cell spread or metastasis in immunocompromised cancer patients.

Stem cells have powerful regenerative and repairing characteristics, which may be employed to repair therapy-associated immune system damage. It is well known that the stem cells in cord blood have strong regenerative and repairing properties, approximately 10 to 20 times what is seen in adult blood [10]. At the same time, stem cells or hematopoietic precursor cells in cord blood also secrete a variety of cytokines, which further promote the repair of damaged tissues and cells [11, 12]. Presently, stem cells in cord blood are mainly used for the replacement of bone marrow hematopoietic stem cells in transplantations for the treatment of hematologic malignancies and hereditary diseases [13]. To our knowledge, there is little information on the use of cord blood for improving immunity in patients with malignant diseases. Currently, numerous drugs and medications have been applied to improve immunity in cancer patients [14, 15], but the overall outcome is disappointing. In this clinical trial, we aim to restore the immunity impaired by tumors or intense chemotherapy and/or radiotherapy in patients with digestive system malignances by transfusing allogeneic umbilical cord blood mononuclear cells (UCMCs). In comparison with control patients, we investigated the effects of allogeneic UCMC transfusion on the immunity of immunosuppressed cancer patients.

## 2. Patients and Methods

### 2.1. Patients and Clinical Design

This clinical research was approved by the Chinese Health Ministry [Health Management Act (2009) number 84]. Before starting the project, this was approved by the Ethics Committee of the participating hospitals, and a written consent from each patient was obtained in accordance with the 1980 Declaration of Helsinki. A total of 130 patients diagnosed as having different gastrointestinal cancers at stage II or III were enrolled in the study from the Affiliated Hospital of Inner Mongolia Medical University and the Ordos Rehabilitation Hospital between October 2009 and October 2015. The inclusion criteria included a Karnofsky score of 60 or greater and hematological parameters before treatment (hemoglobin > 9.0 g/dL; total granulocyte count > 2000/*μ*L; platelet count > 80,000/*μ*L; blood urea nitrogen [BUN] < 30 mg/dL; creatinine < 2 mg/dL; serum bilirubin < 400 *μ*mol/L; alkaline phosphatase and aspartate aminotransferase less than twice the upper limit of normal; prothrombin time and activated partial thromboplastin time of no greater than 1.4 times control values).

Key exclusion criteria included the following: KPS < 60%, cachexia, being HIV positive, drug addiction, pregnancy, severe pulmonary or cardiac diseases, superior vena cava syndrome, ileus, blood transfusion within 12 weeks of the study, evidence of active tuberculosis, and uncontrolled diabetes.

The patients were randomly assigned to UCMC transfusion and no-transfusion groups. Randomization was based on a centrally designed randomization table. All the patients had completed conventional treatments (radiotherapy and/or chemotherapy) at least 1 week before the start of this clinical trial. The patients' immunity was measured with flow cytometry and ELISA.

### 2.2. Conditioning and Transfusion Procedure

All patients in the UCMC transfusion group received a nonmyeloablative (NMA) conditioning regimen. The reduced NMA regimen consisted of cyclophosphamide 25 mg/kg (day 6), fludarabine 20 mg/m^2^ for 5 days (from day 6 to day 2) after routine treatments for gastrointestinal cancers. However, the patients in the control group did not go through NMA.

**Figure 1:**
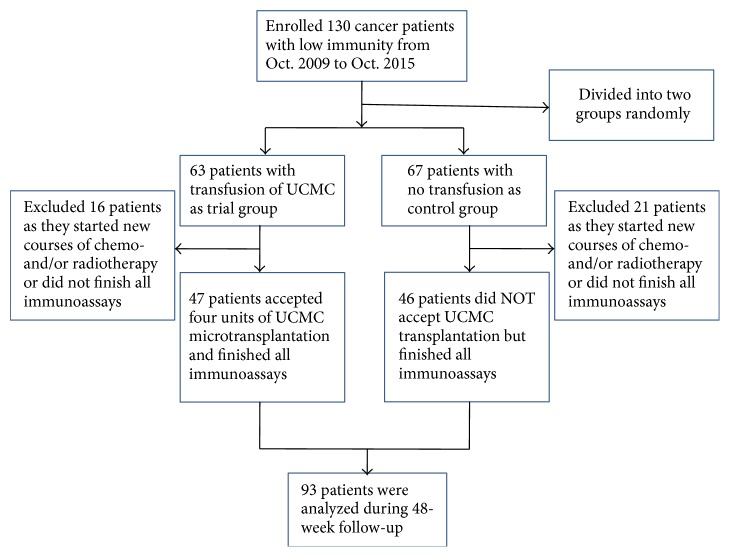
CONSORT diagram of patient distribution. A total of 37 patients were excluded from the clinical data, including 16 in the trial group and 21 in the control group, because these patients did not finish all necessary immunoassays or accepted new courses of radiotherapy and/or chemotherapy during the 48-week follow-up.

ABO matched and partially HLA matched (≥4 alleles) allogeneic UCMCs were transfused to 63 cancer patients with a NMA conditioning regimen. Sixty-seven patients in the control group were not given UCMCs. The distribution of patients in the clinical study is depicted in Figure 1. Blood samples were generally obtained before NMA and at 4, 8, 12, 16, 24, 36, and 48 weeks after treatment for immunity analysis. Clinical characteristics of patients are summarized in Table 1. Any patients that started new courses of chemo- and/or radiotherapy during the follow-up period or missed necessary immunoassays were excluded from this study.

### 2.3. Umbilical Cord Blood Mononuclear Cells (UCMCs)

The UCMCs were provided by the Shandong Jinan Qilu Umbilical Cord Hematopoietic Stem Cell Bank and separated in the Inner Mongolia Blood Bank. All the umbilical blood samples were tested for HIV1/2, HBV, HCV, syphilis, and other common contaminants. ABO and HLA typing had been done before the cells were stored in the banks. Cells with compatible ABO and that matched at least four HLA alleles were used for transfusion. The viability of resuscitated UCMC was 94% to 98%. Four units of UCMCs were transfused to 63 patients (mononuclear cells: 10.0–15.0 × 10^8^/unit). Each unit of UCMC was transfused to the trial patients every week for 4 consecutive weeks.

### 2.4. Monitoring Chimerism after HLA-Mismatched UCMC Transfusion

Chimerism was monitored once for each patient at week 48 after the last UCMC transfusion. Peripheral blood mononuclear cells (PBMCs) from cancer patients transfused with HLA-mismatched UCMC were monitored for autologous cells using HLA-specific antibodies in combination with lineage specific antibodies [16]. FITC-labeled HLA-serotypes, A2/28, A3, A9, B7/27, B8, B12, B27, and Bw4 or Bw6, were used. All the HLA typing was done in the Shandong Jinan Qilu Umbilical Cord Hematopoietic Stem Cell Bank.

### 2.5. Quantification of Lymphocyte Subsets

Peripheral blood samples were collected after conventional treatments (between routine treatments and NMA) and at 4, 8, 12, 16, 24, 36, and 48 weeks after the last transfusion of UCMCs. The samples were analyzed by flow cytometry using a 5- or 7-reagent panel of fluorochrome-conjugated monoclonal antibodies specific for the following surface antigens: CD3-FITC, CD4-APC, CD8-PE, CD19-FITC, CD25-PE, FoxP3-FITC, and CD16/CD56 (BD Biosciences). After incubation with monoclonal antibodies, red blood cell lysis was performed using BD Pharm Lyse. Flow cytometry was performed on a FACS Canto II (BD Bioscience) and analyzed using BD FACSDiva software.

### 2.6. Serum Cytokine Measurement

Serum levels of cytokine IFN*γ*, TNF*α*, and IL10 were quantified with ELISA kits (MLBIO, Shanghai) before and at different intervals after UCMC transfusion.

### 2.7. Clinical Responses

The following response evaluation criteria in solid tumors (RECIST) were used: CR (complete response), disappearance of all target lesions; PR (partial response), 30% decrease in the sum of the longest diameters of target lesions; PD (progressive disease); 20% increase in the sum of the longest diameters of target lesions; SD (stable disease); and small changes that did not meet the above criteria.

### 2.8. Statistical Analysis

The Wilcoxon rank-sums test was used to compare immune recovery parameters at approximately 1 day before and at weeks 4, 8, 12, 16, 24, 36, and 48 after the last transfusion. Pearson's Chi-square test of proportions was used to compare associations between patients' disease status and UCMC transfusion.

## 3. Results

### 3.1. Characteristics of Patients

Sixty-three cancer patients were treated with transfusion of UCMC from an HLA partially matched unrelated donor with NMA conditioning regimen as a trial group, and 67 patients with no UCMC transplantation were the control group. All the cancer patients had completed cytotoxic chemo- and/or radiotherapy and finished all necessary immunoassays before UCMC transfusion. The detailed descriptions of these patients are shown in Table 1. There was no significant difference in the age of UCMC recipients (mean age 55.4; range 23–80 years) and that of control patients (mean age 56.3; range 27–77 years) (*p* > 0.05). In addition, there were no significant differences in the underlying malignancies and treatment methods between the two groups (*p* > 0.05).

### 3.2. Chimerism after UCMC Transfusion

The patients were analyzed at week 48 after UCMC transfusion for chimerism in peripheral blood. Fluorescence intensity of HLA antibodies against the same HLA-antigen differed among patients. Therefore, both donor and recipient pretransfusion samples were analyzed for baseline fluorescence levels. Posttransfusion sample fluorescence intensity is then compared with the baseline levels. In all cases, HLA differences and antibodies could be found to distinguish between donor and recipient. Flow cytometry analysis showed that there was no detectable chimerism in all tested patients 48 weeks after UCMC transfusion.

### 3.3. Lymphocyte Alteration

To assess the impact of UCMC transfusion on the recovery of specific lymphocyte subsets, we measured various CD markers of lymphocytes at different intervals after UCMC transfusion. Blood samples were collected at the same time points in the control group as in the transfusion group. We analyzed the numbers of CD3+, CD3+CD4+, CD4+Foxp3, CD3+CD8+, CD16+CD56+, and CD19+ cells, as well as the ratios of Foxp3+/CD4+(Treg/CD4+) and CD4+/CD8+, using bivariate statistics. It showed that UCMC transfusion promoted lymphocyte subsets, increasing to normal range within 12 weeks after 4 units of UCMC transfusion in 68.1% (32/47) patients, and sustained them at a stable level during 48-week follow-up. In the control group, however, the proportion of patients with automatic immunity recovery to normal level was 26.1% (12/46) at the same time intervals (4 weeks plus 12 weeks = 16 weeks) (*p* < 0.01).

### 3.4. T Cell Recovery

#### 3.4.1. CD3+ Cells

**Figure 2:**
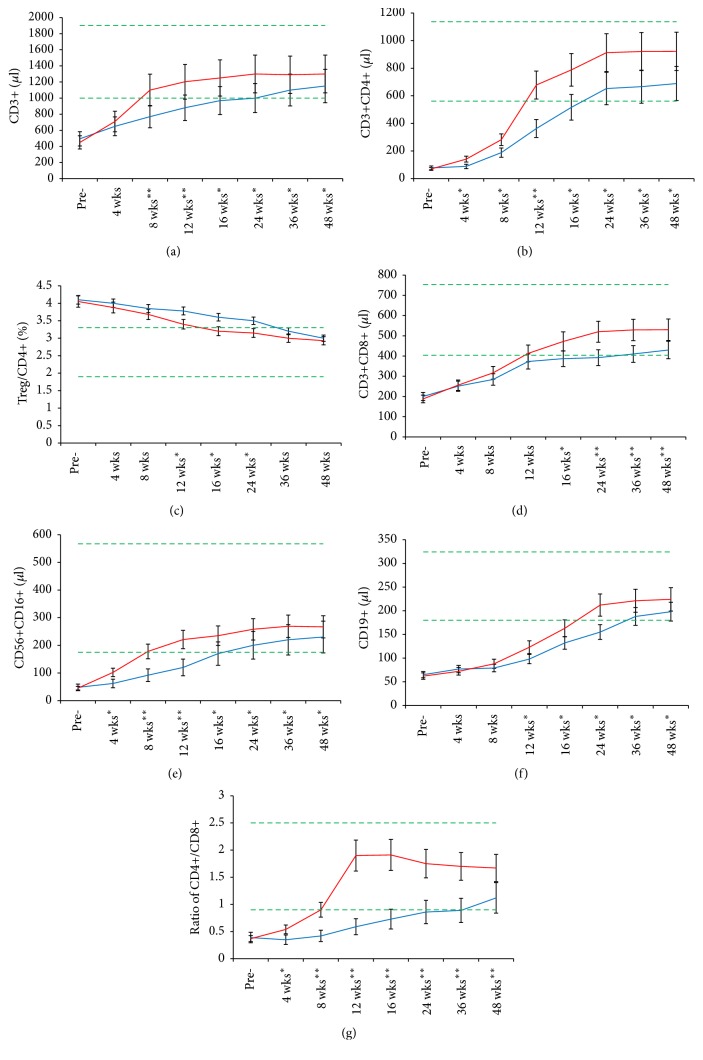
Sequential changes of lymphocyte subsets with flow cytometry analysis. Pre-: before NMA; 4, 8, 12, 16, 24, 36, and 48 wks: after UCMC transfusion. (a)* CD3+T cell; *(b) CD3+CD4+T helper; (c) ratio of Treg/CD4+; (d) CD3+CD8+ cytotoxic T cell; (e) CD16+CD56+NK cell; (f) CD19+ B cell; (g) ratio of CD4+/CD8+. The reference range is depicted as that between the green dot lines; red solid line: UCMC transfusion; blue solid line: control group. Comparison between the trial and control groups, ^*∗*^*p* < 0.05, ^*∗∗*^*p* < 0.01.

The number of CD3+, CD4+, and CD8+ cells and the ratio of CD4+/CD8+ were significantly lower than the normal range in 89.2% (116/130) of patients who accepted conventional treatments. Normally, 4 units of UCMC transfusion promoted absolute number of CD3+ T cells increasing by more than onefold (Figure 2(a)). The increasing of CD3+ T cells started at week 4 and took about 8 weeks to reach a stable level in 72.3% (33/47) of cancer patients with no further radio- or chemotherapy after the last UCMC transfusion. However, the percentage of patients with CD3+ growing to normal level was 23.9% (11/46) in the control group 12 weeks (4 wks + 8 wks) after conventional treatments (*p* < 0.01).

#### 3.4.2. CD4+ Cells and Treg Cells (CD4+Foxp3+)

Over 90% (118/130) of patients had a lower-than-normal range of CD4+ after chemo- and/or radiotherapies. The mean time for CD4+ T cell reconstruction to normal range after UCMC transfusion took about 12 weeks, but the automatic recovery of CD4+ took more than 24 weeks without UCMC transplantation (Figure 2(b)).

CD4+ T cells were more sensitive to chemo- and/or radiotherapies than were Treg cells. Therefore, the ratio of Tregs to CD4+ T cells was higher than the normal range in 74.6% (97/130) of cancer patients.

Although the ratio of Tregs to CD4+ T cells was in the higher-than-normal range in most gastroenteric cancer patients, the absolute number of Tregs was lower than normal references in 91.5% (119/130) of patients. The ratio of Treg/CD4+ started decreasing at week 8, reaching the normal range at week 16 after UCMC transfusion, but it took more than 36 weeks to recover to normal level in the control group (Figure 2(c)).

#### 3.4.3. CD8+ T Cells and Ratio of CD4+/CD8+

Compared with CD4+, CD8+ T cell growth was slower, as it increased to the normal reference range at week 12 after UCMC transfusion. This difference was statistically significant (Figure 2(d)) (*p* < 0.05). The recovery of the CD4+/CD8+ ratio started at week 4 after transfusion and reached a peak at week 12 (Figure 2(g)), which was in accordance with the recovery of CD4+ T cells. However, CD8+ T cell automatic recovery to normal reference level took more than 24 weeks in 45.6% (21/46) of cancer patients in the control group.

#### 3.4.4. NK Cells

NK cells were extremely sensitive to numerous cytotoxic chemotherapeutic drugs and radiotherapy, the reason for which is that the absolute number decreased to less than one-third of normal level after one course of therapy in 92.3% (120/130) of patients. Similar to that of T cells, the populations of CD16+CD56+ NK cells with both NK and T cell markers started growing at week 4 and reached up to 5-fold growth at week 8 after UCMC transfusion in 72.3% (34/47) of patients. The cell number stayed at a stable level during the follow-up period (Figure 2(e)). However, the cells growing to normal range took over 16 weeks in 76.1% (35/46) of patients in the control group.

This clinical investigation showed that the absolute number of CD19+ B cells decreased after chemo- and/or radiotherapies in 72.3% (94/130) of patients. In contrast to that of T lymphocytes, the recovery of CD19+ B cells was slower, as it began at week 12 after UCMC transfusion (*p* < 0.05). Remarkably, the absolute number of CD19+ B cells increased up to 4-fold and sustained the same level from week 24 to the end of the 48-week follow-up period as long as no further chemo- and/or radiotherapies were imposed in 87.2% (41/47) of patients in the trial group. However, the CD19+ B cell recovery in the control group was slower than that of the UCMC transfusion group (Figure 2(f)) (*p* < 0.05).

### 3.5. Soluble Cytokine Production

**Figure 3:**
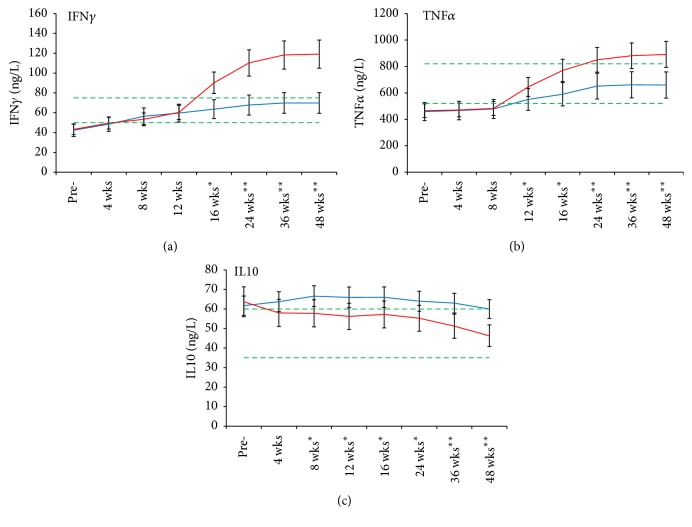
Sequential changes of cytokines during follow-up. The reference range is depicted as that between the green dotted lines; red solid line: UCMC transfusion; blue solid line: control group. Comparison between the trial and control groups, ^*∗*^*p* < 0.05 and ^*∗∗*^*p* < 0.01.

Proinflammation cytokines IFN*γ* and TNF*α* in serum were lower than normal reference range in 87 of 130 (66.9%) cancer patients after chemo- and/or radiotherapies. The concentration of them increased to or above the normal range in 38 of 47 (80.9%) patients at 12 weeks after UCMC transfusion. However, they recovered to the normal reference range in only 10 of 46 (21.7%) patients at the same time point in the control group. Statistical comparison showed that the two groups had significant differences during the 48-week follow-up (Figures 3(a) and 3(b)) (*p* < 0.01).

In contrast, anti-inflammation cytokine IL10 was higher than the normal range in 98 of 130 (75.4%) patients. UCMC transfusion suppressing IL10 production started from week 4 and remained within the normal reference range in 36 of 47 (76.6%) patients during the 48-week follow-up; however, only 8 of 46 (17.4%) patients decreased to normal range in the control group (*p* < 0.01) (Figure 3(c)).

### 3.6. Patients' Status during Follow-Up

No serious side effects were noted in patients transfused with allogeneic UCMCs. The distribution of cancer patients is shown in Figure 1. A total of 37 patients were excluded from the clinical data, including 16 in the trial group and 21 in the control group, because these patients did not finish all necessary immunoassays or accepted new courses of chemo- and/or radiotherapies during the follow-up period. To investigate the effects of UCMC transfusion on disease status, we evaluated the patients' condition every 12 weeks. The rates of CR, PR, SD, PD, and mortality are shown in Table 2 in the two groups at the end of follow-up. Statistical analysis indicated that UCMC transfusion had no significant effects on CR, PR, and mortality (*p* > 0.05) but improved SD and reduced PD significantly (*p* < 0.01) (Table 2).

## 4. Discussion

The immune system plays a key role in the body's own defenses against cancer cells. Malignancy invariably leads to immunocompromise in patients by natural disease effects. Cytotoxic chemo- and/or radiotherapies cause severe hematopoietic inhibition and immunity suppression. The present clinical investigation confirmed that conventional cancer therapies suppressed cancer patients' immunity significantly. One course of chemotherapy can kill more than 50% of CD3+ cells, aggravating immunosuppression. A long-term immunocompromised condition in cancer patients increases cancer recurrence and metastases [17]. Numerous techniques and drugs have been tried to enhance immunity in cancer patients [18, 19], but no one of them has been confirmed clinically to promote immunity in cancer patients.

In this clinical study, we confirmed that allogeneic UCMC transfusion with a NMA conditioning regimen achieved powerful restoration of lymphocytes in cancer patients with immunosuppression. After UCMC transfusion, the restoration of CD8+ cells was slower than that of CD4+ cells. The mechanism of time difference in restoring the two types of immune cells is not clear. NK cells are very sensitive to chemotherapy. Their number was reduced to less than 50% of the original value in most cancer patients after receiving one course of cytotoxic chemotherapy, but their recovery was rapid starting from 4 weeks after UCMC transfusion. The reason for quick restoration might be that there are abundant NK precursors CD16+CD56− in the UCMCs [20], and they differentiate to mature NK cells after transfusion. Although our data did not support this mechanism as chimerism, we cannot exclude possible chimerism at the early stage of transfusion, as the transfused allogeneic cells may have been rejected after 48 weeks. Another possible reason is UCMC's secret lymphokines or stem cell factors, which promote cell proliferation and repair injured or damaged NK cells [21].

The function of CD4+CD25+Foxp3+Tregs in cancer had been described as highly variable [8, 22]. In order to assess the impact of UCMC transfusion on cellular immunity recovery, we measured the absolute number of Tregs and its ratio to CD4+ T cells. We also compared Treg recovery in the two groups. In contrast to other lymphocyte subsets, the ratio of Tregs to CD4+ T cells after chemotherapy increased because the decrease in CD4+T cell was more severe than that of Tregs. It should be noted that the ratio of Tregs to CD4+T during 48-week follow-up remained higher than the normal value in the control group, although the absolute number was lower than normal range, which was consistent with a previous study [23]. Higher ratio of Tregs means lower immunity in cancer patients.

Our study confirmed that the immunity of cancer patients is severely damaged by intensive chemo- and/or radiotherapies. Because B lymphocyte maturation and marker expression require assistance from T lymphocytes, the damaged T lymphocytes are generally repaired first. T cells, on full recovery, can assist in the repairing and functioning of B cells [24]. With respect to the dependency of B cells on T lymphocytes, it is possible to explain the reason for slower recovery of B lymphoid cells than that of T cells.

Chemo- and/or radiotherapies have significant effects on the production of soluble cytokines in serum. This clinical study confirmed that cancer patients have elevated anti-inflammation cytokine after conventional therapies [25, 26].

UCMC could enhance proinflammation cytokine and suppress anti-inflammation cytokine production. The possible explanations are that UCMC transfusion promoted T helper and natural killer cell recovery and suppressed Treg directly, or unknown factors secreted by UCMC promoted T cell activation to release proinflammation cytokines [27–29].

It is of interest to note that, despite the observed quick recovery in quantitative T cell types in recipients of UCMC, we did not observe significant differences in the rates of CR, PR, and mortality compared with the control group. However, UCMC transfusion did significantly increase SD and reduce PD during the 48-week follow-up. This can be explained, in part, by the improvement in cellular immunity.

Although restorative activity of UCSC played an important role in the immunity recovery, the reaction of the patients' immune system to allogeneic cells may play a part as well, which is difficult to be proved in this clinical study; more research is needed in the future.

## 5. Conclusions

Unrelated umbilical cord blood mononuclear cell transfusion could enhance immunity of immunocompromised cancer patients. The immunity improvement could increase the rates of SD and decrease PD in cancer patients but did not have significant effects on rates of CR, PR, and mortality.

## Figures and Tables

**Table 1:** Patients' characteristics.

Characteristics	UCMCs transfusion	No-transfusion	*p* value
Number of patients	63	67	
Age (years), mean (range)	55.4 (23–80)	56.3 (27–77)	>0.05
Sex			>0.05
Female	21 (33.3%)	27 (40.3%)	
Male	42 (66.6%)	40 (59.7%)	
Diseases			>0.05
Gastric lymphoma	2 (3.2%)	2 (3.0%)	*∗*
Gastric adenocarcinoma	29 (46.0%)	29 (43.3%)	
Colon adenocarcinoma	24 (38.1%)	26 (38.8%)	
Rectal carcinoma	7 (11.1%)	10 (14.9%)	
Langerhans cell sarcoma in small intestine	1 (1.6%)	0 (0%)	*∗*
History of previous treatments			>0.05
Chemotherapy only	19 (30.2%)	22 (32.8%)	
Radio- & chemotherapy	9 (14.3%)	11 (16.4%)	
Surgery & chemotherapy	34 (54.0%)	32 (47.8%)	
Surgery & radiotherapy	1 (1.6%)	2 (3.0%)	

^*∗*^No statistical test is provided due to small sample size.

**Table 2:** Patients' status during follow-up.

Patients' status	UCMC (47 patients)	Control (46 patients)
CR	10.6% (5/47)	13.0% (6/46)
PR	14.9% (7/47)	15.2% (7/46)
SD	44.7% (21/47)^*∗∗*^	21.7% (10/46)
PD	19.1% (9/47)^*∗∗*^	41.3% (19/46)
Died	10.6% (5/47)	8.7% (4/46)

CR, complete response; PR, partial response; SD, stable disease; PD, progressive disease. ^*∗∗*^*p* < 0.01.
